# Retraction: The role of *CRP* and *ATG9B* expression in clear cell renal cell carcinoma

**DOI:** 10.1042/BSR-2017-1082_RET

**Published:** 2023-06-28

**Keywords:** ATG9B, cell apoptosis, clear cell renal cell carcinoma (CCRCC), CRP

This Retraction follows an Expression of Concern relating to this article previously published by Portland Press. This article is being retracted from *Bioscience Reports* at the request of the Editor-in-Chief and the Editorial Board following receipt of a notification from a reader, alerting the Editorial Board to images that appear similar between this paper and those by unrelated authors.

Specifically, the Figure 3A control panel appears similar to the Figure 4A UA panel from Li et al. 2017 (doi: 10.18632/oncotarget.22133). The Figure 4B GAPDH western blot bands also appear similar to the Figure 12D A549/PTX/B-actin bands from Su et al. 2018 (doi: 10.1002/cam4.1294) and the Figure 2E SCC-9/GAPDH bands from Wang et al. 2019 (doi: 10.1016/j.biopha.2017.04.050).

The authors have been contacted with regards to the retraction and have not responded to the Journal's queries or the concerns raised. Given the extent of the issues raised, the Editorial Board stand by the decision to retract the article.

